# Ultrasound Evaluation of the Regenerating Tendon of the Semitendinosus After Harvest for Anterior Cruciate Ligament Reconstruction

**DOI:** 10.3390/jcm14217862

**Published:** 2025-11-05

**Authors:** Marco Becciolini, Michele Bisogni, Salvatore Massimo Stella, Carlo Trompetto, Laura Mori, Luca Puce, William Campanella, Orlando Catalano, Filippo Cotellessa

**Affiliations:** 1 Misericordia di Pistoia, Via Bonellina 1, 51100 Pistoia, Italy; 2 Scuola Siumb di Ecografia Muscolo-Scheletrica, 56128 Pisa, Italy; 3 Centro Medico Restart, 53100 Siena, Italy; 4 Department of Neuroscience, Rehabilitation, Ophthalmology, Genetics, Maternal and Child Health, University of Genova, 16126 Genova, Italyfilippo_cotellessa@hotmail.it (F.C.); 5 IRCCS Ospedale Policlinico San Martino, 16132 Genoa, Italy; 6 Radiology Unit, Istituto Diagnostico Varelli, 80126 Napoli, Italy

**Keywords:** ultrasound, dynamic, tendon, regeneration, ACL

## Abstract

**Objectives**: Previous studies have suggested the potential for semitendinosus (ST) tendon regeneration following harvesting for anterior cruciate ligament (ACL) reconstruction. This retrospective cross-sectional (observational) study aims to evaluate ST tendon regeneration using high-resolution ultrasound (US), with special reference to morphological and structural changes in the muscle–tendon unit. **Methods**: Twenty-four patients who had undergone ST tendon harvesting at least 24 months prior were evaluated. Ultrasound assessment included neotendon (NT) detection, thickness, echotexture, insertion site, dynamic gliding, myotendinous junction (MTJ) shifting, and muscle cross-sectional area (CSA), compared with the healthy contralateral side. **Results**: ST tendon regeneration was detected in 19/24 patients (79%). In regenerated tendons, NT thickness was significantly greater than the native tendon (3.40 ± 1.38 mm vs. 2.40 ± 0.27 mm; mean difference 0.98 mm; *p* = 0.005. Subgroup analysis revealed that fibrillar-like NTs were associated with a smaller MTJ shift (3.91 ± 1.14 cm vs. 7.75 ± 2.43 cm; *p* = 0.001) and higher muscle CSA preservation (0.85 ± 0.10 vs. 0.55 ± 0.09; *p* < 0.001). A strong inverse correlation was found between MTJ displacement and muscle CSA (ρ = −0.96; *p* < 0.001). Patients without NT regeneration (n = 5) exhibited more pronounced MTJ retraction (11.0 ± 1.0 cm) and muscle hypotrophy (CSA ratio 0.41 ± 0.07), although these results were descriptive. **Conclusions**: High-resolution US is an effective, non-invasive method for assessing ST tendon regeneration from a qualitative and quantitative perspective. Our findings indicate possible changes in the architecture and position of the regenerated tendon, the MTJ, and the muscle belly, which may reflect structural remodeling of the muscle–tendon unit.

## 1. Introduction

**Figure 1:**
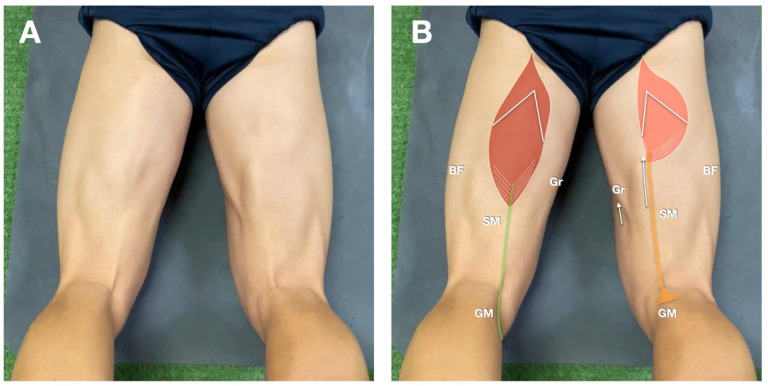
(**A**) Clinical photo of the posterior thigh in a study participant during knee flexion against resistance. The right knee is the operated side. (**B**) Same image with schematic drawings of the ST tendon of the patient’s left healthy side (in green), the ST neotendon (in orange). The image also shows the ST muscle and myotendinous junction, with retraction on the affected side (white arrow). Retraction also involves the gracilis muscle (Gr). Note that this image will be used as a reference for probe positions. Please refer to this legend for abbreviations. BF indicates the biceps femoris; GM the gastrocnemius medialis; SM the semimembranosus.

Tendon regeneration following semitendinosus (ST) harvesting for anterior cruciate ligament (ACL) reconstruction remains a widely debated topic in the literature [1]. Several studies have reported the potential for partial or complete regrowth of the ST tendon, with variable structural organization and biomechanical functionality (Figure 1) [2,3,4,5]. One of the most critical issues is whether this “neo-tendon” (NT) can restore the biomechanical characteristics of the native tendon and how this influences the function of the ST muscle [6]. Prior studies have highlighted substantial variability in regeneration quality, with findings that include muscle hypotrophy, changes at the myotendinous junction (MTJ), and reduced NT gliding compared to the healthy contralateral side [7,8,9,10]. However, a study that evaluates extensively the morphology and dynamic behavior of the ST muscle–tendon unit is lacking, underscoring the need for further investigation.

From a clinical perspective, understanding the morphology of the regenerated tendon is relevant. The ST contributes to knee flexion and internal rotation, and its harvest may influence muscle strength, joint stability, and long-term outcomes. Assessing whether and how the tendon regenerates and how the muscle changes with respect to the normal contralateral side could, therefore, help clarify the causes of persistent weakness or altered biomechanics observed in some patients after ACL reconstruction. Moreover, reliable imaging of the NT may support clinicians in tailoring rehabilitation strategies and monitoring recovery.

High-resolution ultrasound (US) has emerged as a reliable imaging modality for assessing the morphological and functional features of the regenerated ST. This technique enables detailed analysis of the newly formed tissue’s echotexture and dynamic behavior [11,12]. Moreover, US is more widely accessible than MRI. This study aims to assess ST tendon regeneration after harvesting for ACL reconstruction using US. Specifically, this research aims to evaluate by imaging outcomes, the NT thickness, structure, and sliding capacity, as well as changes in the MTJ and ST muscle belly. The goal is to improve our understanding of the morphological outcome of the ST muscle–tendon unit after ACL harvesting, at least two years after surgery, when remodeling processes have ended.

## 2. Materials and Methods

### 2.1. Study Design and Participants

This retrospective cross-sectional (observational) study was conducted to evaluate the ultrasonographic characteristics of regenerated ST following harvest for anterior cruciate ligament reconstruction. A total of 24 patients (mean age at surgery: 29 years; range: 14–64 years; M:F ratio = 21:3) who had undergone tendon harvesting at least 24 months prior to the US evaluation were included. Exclusion criteria included bilateral ACL reconstruction, clinical or radiological evidence of contralateral ST tendinopathy, and a history of distal hamstring injury or rupture.

The study protocol was reviewed and approved by the local ethical committee (protocol number 2025.51).

### 2.2. Ultrasonographic Assessment

All patients underwent bilateral ultrasound examination of the ST tendon and muscle using a Canon Aplio 300 (Canon Medical Systems, Otawara, Japan) equipped with linear multi-frequency high-resolution transducers (14–5 MHz, with a field of view width of 58 mm; and 18–7 MHz, with a field of view width of 25 mm).

### 2.3. Tendon Regeneration, Morphometry, and Echostructural Features

**Figure 2:**
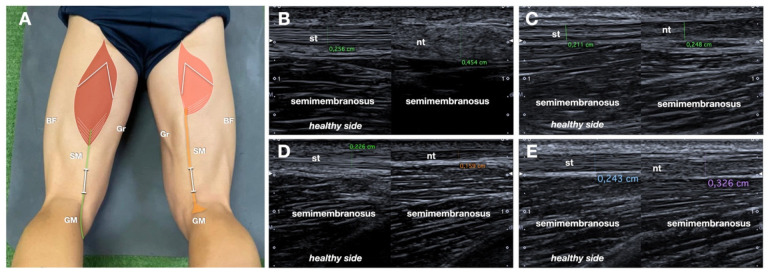
Neotendon thickness and echostructure in different patients. (**A**) Schematic drawing (same as Figure 1B, refer to it for the abbreviations) indicating probe position. (**B**–**E**) Longitudinal comparative sonograms at the distal third of the thigh demonstrate the neotendon thickness (indicated in cm, measured also on the healthy side) and echostructure. (**B**) Iso-hyperechoic, non-fibrillar. (**C**) Hypoechoic, non-fibrillar. (**D**) Hypoechoic, fibrillar-like. (**E**) Iso-hyperechoic, fibrillar-like. nt indicates the neotendon; st, semitendinosus tendon.

Regeneration of the NT was assessed based on the presence of a continuous tendon-like structure in the expected anatomical course, near the posterior aspect of the knee [13]. Thickness (mm) and echotexture characteristics of the regenerated tendon were assessed in the longitudinal plane at a standardized distal-thigh level and compared to the contralateral, unharvested ST tendon (Figure 2).

Echostructure of the regenerated tendon was classified into four categories based on the grayscale appearance: (1) fibrillar-like hypoechoic, (2) fibrillar-like iso/hyperechoic, (3) non-fibrillar hypoechoic, and (4) non-fibrillar iso/hyperechoic. The term “fibrillar-like” was employed to describe tendons displaying linear echogenic strands mimicking a normal fibrillar pattern, though not identical to native morphology, with hyperechoic non-fibrillar foci, consistent with fibrotic scarring. Non-fibrillar was used for more disorganized NT, without the typical aforementioned fibrillar pattern. The echostructure was defined as hypoechocic or iso/hyperechoic in comparison with the normal ST. Tendon gliding during active knee flexion-extension was assessed dynamically in real time (Video S1). This dynamic sonogram was obtained with the transducer in the long axis of the NT (transducer placed as shown in Figure 2), with the patient in prone position, starting with knee extension, to reach 90° flexion. To ensure the tendon gliding was correctly depicted, the patients were asked to do three flexion-extension repetitions.

### 2.4. Distal Insertion Site

**Figure 3:**
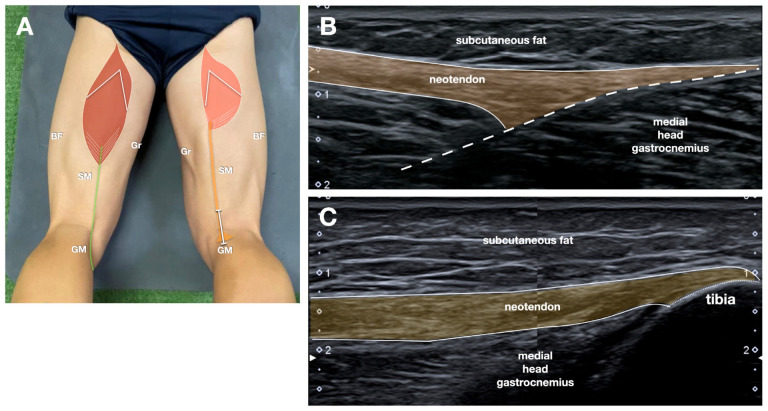
Neotendon insertion. (**A**) Schematic drawing (same as Figure 1B, refer to it for the abbreviations) indicating probe position. (**B**) In most of the patients, the neotendon (highlighted in yellow) showed an attachment directly to the medial head of gastrocnemius superficial fascia (dotted line). (**C**) In 3 patients, the neotendon was inserted into the tibia.

The distal insertion of the regenerated NT was evaluated to determine whether the tendon reinserted into the native pes anserinus region or deviated toward alternative fascial planes, including the gastrocnemius or semimembranosus fascia (Figure 3).

### 2.5. Myotendinous Junction (MTJ) and Muscle Belly Evaluation

**Figure 4:**
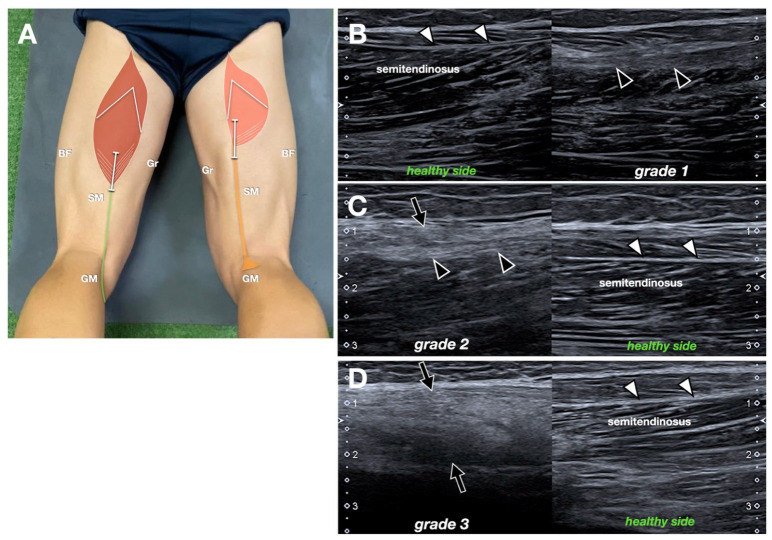
Myotendinous junction. (**A**) Schematic drawing (same as Figure 1B, refer to it for the abbreviations) indicating probe position. (**B**–**D**) Longitudinal comparative sonogram obtained in three different patients. In (**B**), the intramuscular tendon (black arrowheads) is a bit thickened with respect to the normal healthy side (white arrowheads). This was graded as 1. In (**C**), Grade 2, note also the hyperechogenicity of the semitendinosus muscle (black arrow), consistent with mild fatty infiltration, as well as the alteration to the intramuscular tendon (black arrowheads). In (**D**), Grade 3, the muscle is diffusely hyperechoic (black arrows) with partial acoustic shadowing, related to muscular hypotrophy. The intramuscular tendon is no longer recognizable.

The location of the MTJ of the ST was identified as the point of transition from muscle fibers to tendon. Cranial displacement of the MTJ was measured relative to the contralateral limb. Echotexture grading of the MTJ was subjectively defined on a scale from 0 to 3, with the normal healthy side taken as reference (Grade 0, normal muscle). Grade 1 indicated mild hyperechogenicity within the muscle belly. Grade 3 reflected diffuse hyperechogenicity and posterior acoustic attenuation (advanced muscle atrophy) (Figure 4).

**Figure 5:**
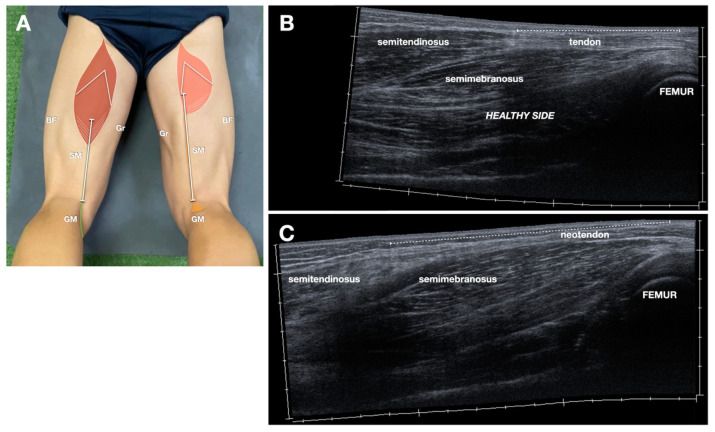
Myotendinous junction shift. (**A**) Schematic drawing (same as Figure 1B, refer to it for the abbreviations) indicating probe position. (**B**,**C**) Extended field of view (panoramic) imaging of the distal semitendinosus, using the posterior medial condyle of the femur as the distal reference. The myotendinous junction is indicated by the calipers. (**B**) The normal healthy side; (**C**) the side of the ST harvest, where the myotendinous junction has shifted proximally (between calipers). Note the difference between the two sides.

An extended field-of-view scan in the long axis of the ST complex of both sides, extending to the level of the distal femoral condyle, was used to measure a potential retraction of the MTJ of the affected side, compared to the healthy limb (Figure 5).

**Figure 6:**
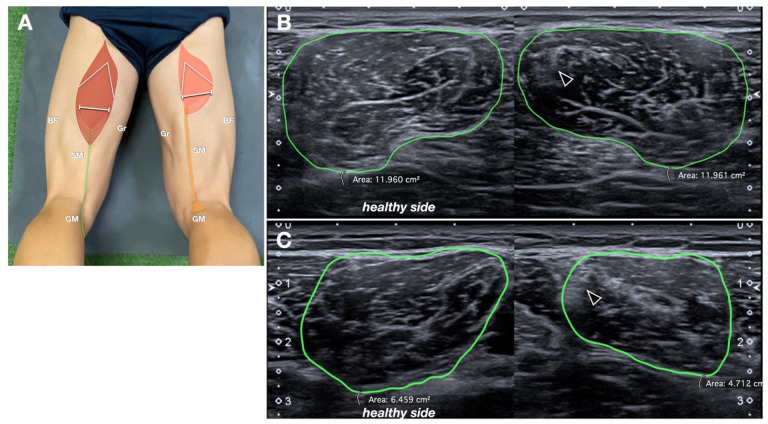
Semitendinosus muscle CSA ratio. (**A**) Schematic drawing (same as Figure 1B, refer to it for the abbreviations) indicating probe position. (**B**,**C**) Comparative axial sonograms in two different patients. The margins of the semitendinosus muscles are manually traced (in green) to obtain the corresponding CSA. In (**B**), the CSA is similar between the two sides, with only some mild echostructural alteration along the intramuscular tendon (black arrowhead) of the semitendinosus muscle. In (**C**), the CSA is reduced more evidently.

The cross-sectional area (CSA) of the ST muscle belly was measured bilaterally, at the middle third of the thigh, using axial sonograms, with measurements compared to the contralateral side (Figure 6). To ensure reproducibility, the CSA was measured at the level where the intramuscular raphe of the ST, a reliable anatomical US landmark for the identification of the muscle, was no longer visible (just caudal to it) [14]. The muscle CSA ratio was calculated similarly to the tendon CSA ratio.

### 2.6. Statistical Analysis

The sample size was calculated for the primary endpoint, defined as the proportion of favorable semitendinosus tendon regeneration, characterized by the presence of a fibrillar-like neotendon with preserved dynamic gliding on ultrasound at long-term follow-up (mean 22 months, range 12–43 months) after harvesting.

A one-sample proportion test (two-tailed) was performed using G*Power 3.1 with α = 0.05 and power = 0.80. Based on the systematic review by Suijkerbuijk et al. [12] which reported a pooled semitendinosus regeneration rate of approximately 79% beyond one year, the expected proportion was set at *p*_1_ = 0.79 against a minimally acceptable threshold of *p*_0_ = 0.50.

Under these assumptions, the required sample size was n = 21 evaluable patients. Considering a 15% potential attrition rate, the target enrollment was set at 25 participants.

The final study population included 24 patients, consistent with the a priori power analysis and ensuring adequate statistical power for the primary endpoint.

All statistical analyses were performed using Jamovi 2.3.28. Continuous variables were tested for normality using the Shapiro–Wilk test, and descriptive data are reported as mean ± standard deviation (SD) unless otherwise specified.

A paired comparison between neotendon (NT) and contralateral tendon thickness was conducted using a paired *t*-test when the distribution of paired differences was normal; otherwise, a Wilcoxon signed-rank test was applied. Results are presented as mean differences with 95% confidence intervals (CIs) and effect sizes (Hedges’ g for paired samples).

Participants were categorized according to the NT echostructure into fibrillar-like and non-fibrillar groups. Between-group comparisons for myotendinous junction (MTJ) shift and muscle cross-sectional area ratio (M CSA %) were performed using independent *t*-tests (equal or unequal variances determined by Levene’s test) or Mann–Whitney U tests when assumptions for normality were not met. Effect sizes were reported as Hedges’ g for parametric and Cliff’s δ for non-parametric tests.

Associations between continuous variables (MTJ shift, M CSA %, age, years after surgery) were assessed using Spearman’s rank correlation coefficients (ρ). All *p*-values were two-tailed, and significance was set at *p* < 0.05. To control for multiple comparisons, the Benjamini–Hochberg false discovery rate (FDR) procedure was applied.

## 3. Results

Table 1 summarizes the demographic and clinical characteristics of the study cohort.

### 3.1. Echostructural Characteristics and Paired Comparison Between NT and Contralateral Thickness

The echostructural characteristics of the regenerated tendons were heterogeneous. Ten NT (53% of the cases) had a hypoechoic appearance with a fibrillar-like pattern, resembling but not identical to the normal contralateral tendon. One NT (5%) appeared iso/hyperechoic with discernible hyperechoic strands, while three (16%) exhibited a non-fibrillar, hypoechoic pattern. Five NTs (26%) were non-fibrillar and iso/hyperechoic. Dynamic assessment during active knee flexion demonstrated normal tendon gliding in all patients, with the exception of one case in which the NT was adherent and did not glide.

Neotendon (NT) thickness was significantly greater than that of the contralateral tendon (3.40 ± 1.38 mm vs. 2.40 ± 0.27 mm; n = 19). The mean paired difference was 0.98 mm (95% CI 0.34–1.62), with a moderate-to-large effect size (Hedges’ g = 0.70; *p* = 0.005).

### 3.2. Where Does the NT Attach?

Analysis of the distal insertion of the regenerated semitendinosus revealed that, in 15 of the 19 patients with tendon regeneration, the NT blended into the fascia of the medial head of the gastrocnemius muscle, proximal to the native pes anserinus insertion. In three patients, the tendon was inserted near its original anatomical footprint on the tibia, close to the pes anserinus. In one patient—the only case in which the regenerated tendon failed to glide during active movement—the NT was seen inserting into the fascia of the semimembranosus muscle, deviating markedly from the typical anatomical configuration.

### 3.3. Subgroup Analysis (Fibrillar-like vs. Non-Fibrillar Echostructure)

When participants were grouped according to NT echostructure, the fibrillar-like group showed smaller MTJ displacement and greater muscle preservation compared with the non-fibrillar group.

MTJ shift: fibrillar = 3.91 ± 1.14 cm vs. non-fibrillar = 7.75 ± 2.43 cm → Mann–Whitney U, *p* = 0.001, Cliff’s δ = −0.95.

Muscle CSA %: fibrillar = 0.85 ± 0.10 vs. non-fibrillar = 0.55 ± 0.09 → *t*-test, *p* < 0.001, Hedges’ g = 2.92.

These results indicate that a fibrillar-like echostructure is associated with more favorable tendon and muscle remodeling.

### 3.4. Correlation Analyses and FDR Correction

Scheme correlations were performed to explore associations among morphometric and clinical parameters. After correction for multiple comparisons using the Benjamini–Hochberg FDR method, the following results were obtained (Table 2).

These correlations confirm a tight relationship between tendon and muscle recovery parameters and highlight a potential age-related influence on regenerative capacity.

The echostructural integrity of the MTJ was altered in all patients with a gliding NT. None of the cases demonstrated a normal (grade 0) MTJ appearance. Five patients (28%), all with fibrillar-like NT, displayed a grade 1 pattern, characterized by hyperechoic thickening at the junction. Grade 2 changes, consisting of mild hyperechogenicity of the semitendinosus muscle, were seen in seven patients (39%), including four with a fibrillar-like NT. Grade 3 changes, reflecting diffuse hyperechogenicity and posterior acoustic attenuation indicative of advanced muscle atrophy, were noted in six patients (33%). Two of these patients had a fibrillar-like NT, and four had a non-fibrillar NT (Table 3).

### 3.5. Patients Without Detectable Tendon Regeneration

**Figure 7:**
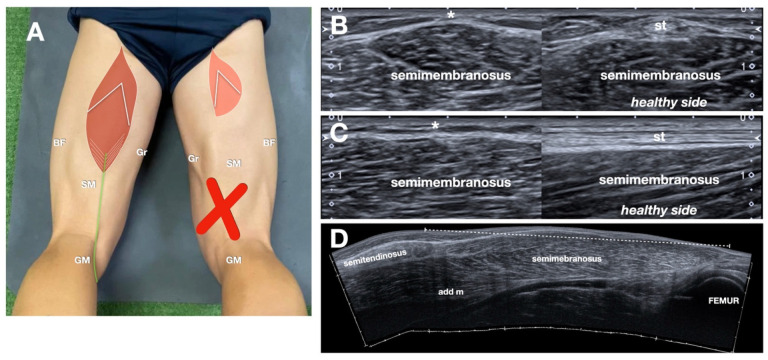
No “regeneration”. (**A**) Schematic drawing (similar to Figure 1B, refer to it for the abbreviations. Note that, in this case, a large red X indicates the absence of an NT) indicating probe position. Note that the patient photo is the same as before; a large red X indicates the absence of an NT. (**B**,**C**) Axial (**B**) and sagittal (**C**) comparative sonograms. Note that, on the left, the absence (asterisk) of the tendon. The normal semitendinosus (st) on the healthy side is demonstrated on the right of the split-screen image. (**D**) Extended field of view (panoramic) imaging demonstrates retraction of the muscle. add m indicates the adductor magnus muscle.

In this subgroup of patients (n = 5) (Figure 7), exploratory descriptive analysis revealed a mean MTJ proximal shift of 11.0 cm (95% CI, 9.7–12.3), consistent grade 3 echostructural changes, and a mean ST muscle CSA ratio of 41% (95% CI, 34–48) relative to the contralateral side. Given the small sample size, these findings should be interpreted as exploratory observations only and are not intended to support generalized conclusions.

## 4. Discussion

The findings of this study provide new insights into the complex process of ST tendon regeneration following ACL reconstruction. The majority of patients (79%) exhibited a regenerated tendon-like structure, although with substantial variability in morphology, insertion site, and structural characteristics.

The observation that most regenerated NT were thicker compared to the contralateral side may be associated with a compensatory hypertrophic response. However, increased size did not necessarily correspond to improved quality. Only a subset of NT showed a fibrillar-like pattern—considered a closer approximation to native tendon structure—whereas a significant number (42%) presented non-fibrillar echotexture. Interestingly, the presence of fibrotic echogenic foci in all NTs suggests that the healing process involves scarring, which may impair elasticity and gliding. These findings are consistent with prior histologic and imaging studies suggesting that tendon regeneration is often incomplete or disorganized, failing to fully replicate the microstructural properties of the original tendon [3,15,16].

Only 3 of 19 regenerated tendons reinserted near the native pes anserinus, while the majority deviated to insert into the gastrocnemius fascia. Although the clinical relevance of this abnormal anatomy is still debated, an aberrantly inserted tendon could suggest a possible association with compromised biomechanics.

Subgroup analysis indicated that fibrillar-like neotendons were associated with smaller MTJ displacement and better muscle preservation compared with non-fibrillar types, suggesting that a more organized regenerative pattern may help limit proximal junctional retraction and secondary hypotrophy. These findings support the concept of a structural–functional continuum, in which tendon quality appears related to the degree of muscle integrity. However, the absence of pre-harvest imaging prevents definitive conclusions about whether these differences reflect true postoperative remodeling or preexisting asymmetries between limbs. Furthermore, variability in surgical techniques, as procedures were performed by multiple surgeons, may have contributed to the heterogeneity in regenerative patterns. Differences in harvesting length, preservation of distal insertions, or fascial handling could have influenced both the morphology of the regenerated tendon and the extent of muscle retraction. Future studies should aim to standardize surgical methods and include baseline imaging to more accurately isolate the determinants of tendon and muscle regeneration.

Correlation analysis confirmed a strong inverse relationship between MTJ displacement and muscle CSA, indicating that greater proximal tendon retraction tended to coincide with lower muscle cross-sectional area. Although this finding does not establish causality, it supports the notion that tendon and muscle remodeling may be interrelated processes. In addition, age showed a positive association with MTJ displacement and a negative trend with muscle CSA, suggesting that older participants may experience less favorable structural recovery. These observations, while exploratory, are consistent with known age-related declines in regenerative capacity and may point to a potential influence of biological aging on post-harvest adaptation of the ST muscle–tendon unit.

Furthermore, echostructural grading of the MTJ consistently showed pathological changes, with grade 3 (indicative of diffuse fibrosis and atrophy) being more present in patients with non-fibrillar NT or absent regeneration. This reinforces the link between tendon morphology and overall musculotendinous health.

The subgroup of patients without detectable tendon regeneration was small (n = 5), and the corresponding results are therefore exploratory and descriptive. While these patients tended to show more pronounced MTJ retraction and muscle hypotrophy, the limited sample precludes any statistical inference or generalization. Future studies with larger cohorts are needed to confirm whether the absence of regeneration consistently is associated with more severe morphological alterations.

Although our study did not directly measure functional outcomes, the imaging findings may offer indirect indications of potential clinical implications. It is plausible that patients showing fibrillar-like NT with preserved gliding could achieve more efficient force transmission and muscle–tendon coordination, possibly contributing to improved strength and functional recovery. In contrast, non-fibrillar or non-gliding tendons, particularly when associated with greater MTJ retraction and muscle hypotrophy, might contribute to persistent weakness, fatigue, or subtle alterations in knee biomechanics reported in long-term follow-up studies. While these hypotheses remain speculative in the absence of direct strength or functional data, the observed imaging patterns appear consistent with known biomechanical principles, suggesting that US may have a role in anticipating functional adaptations after ST harvesting.

US has been demonstrated as an imaging modality to provide reliable measures of tendon thickness and CSA between different operators, so that our results could be reproducible [17,18,19]. Moreover, US may offer advantages over MRI. It provides high-resolution static and dynamic images, allowing for a quick comparison with the contralateral side. It is also more accessible and cost-effective, thus suitable for serial follow-up. MRI offers superior visualization of deeper structures; it is a more panoramic modality that may give a more precise measure of muscle volume (see also the Section 5) and fatty infiltration.

Various studies have explored the regeneration of the ST tendon after harvesting for ACL reconstruction, with highly heterogeneous findings regarding the presence, quality, and functional behavior of the NT. One of the earliest US investigations, conducted by Papandrea et al., reported NT regeneration in all 40 patients evaluated as early as two months postoperatively, with nearly normal echostructure and size documented at 18–24 months [11]. In contrast, our study observed tendon regeneration in only 79% of patients. A possible explanation for this discrepancy lies in the technological evolution of imaging equipment: we employed high-frequency linear probes (up to 18 MHz), which offer superior spatial resolution compared to the 7.5 MHz transducer, in particular for superficial structures, allowing for more accurate discrimination between true tendinous tissue and fibrotic or disorganized structures.

In contrast, the study by Bedi et al. provided a more conservative view of tendon regeneration [3]. Among 18 knees evaluated at an average of one year post-ACL reconstruction, 50% exhibited no visible tissue bridging the harvest gap. Only 4 knees showed a fibrillar-like hypoechoic pattern, and dynamic ultrasound detected no movement in 3 out of 9 knees studied. Interestingly, our results partly mirror these findings, especially in patients with non-fibrillar or non-gliding NT. However, we identified a higher prevalence of fibrillar-like patterns (11/19 NT) and preserved dynamic gliding in all but one case, suggesting that tendon regeneration may progress further with time, especially considering that Bedi included patients evaluated as early as 66 days post-surgery. We evaluated patients at least 24 months post-surgery, which could have allowed more complete tissue remodeling and integration. Moreover, our study applied a more in-depth protocol, allowing for the comprehensive characterization of the ST muscle–tendon unit.

Regarding muscle volume, Karagiannidis et al. reported a paradoxically increased ST muscle thickness in patients post-ACL reconstruction compared to healthy controls [20]. This is in contrast with our results, which showed consistent hypotrophy in the harvested limb, especially in patients with non-fibrillar or absent NT. The discrepancy may be due to methodological bias: Karagiannidis compared different individuals, introducing confounding factors such as rehabilitation intensity, baseline muscle mass, or surgical variability. In our study, we opted for an intra-patient comparison between the operated and contralateral limb, minimizing interindividual variability and better isolating the effects of tendon harvest.

## 5. Limitation

Our study has several limitations that must be acknowledged. First, the sample size is relatively small. Furthermore, the age distribution of our patient population was not homogeneous, potentially affecting tendon and muscle regeneration, as age is a known factor influencing tissue healing and remodeling capacity. Another methodological limitation is the lack of preoperative US evaluation, which would have allowed documenting the baseline condition of the ST before harvest. It prevents confirmation of the baseline comparability of tendon and muscle morphology between the operated and contralateral limbs.

Regarding muscle evaluation, MTJ echotexture was subjectively graded, and we assessed only the CSA at the mid-thigh level, which is a partial indicator of total muscle volume. A more comprehensive volumetric analysis—ideally using MRI—could have provided a more accurate assessment of muscle hypotrophy as shown in Snow’s study, which demonstrated a loss of 40 to 50% in volume [21]. Moreover, recently, a deep model for predicting skeletal muscle density from US has been proposed, which could provide more objective parameters for evaluating the MTJ trophism [22].

Nevertheless, the lack of clinical or functional assessment represents a limitation of our study. Without correlating imaging results with objective performance measures or patient-reported outcomes, it remains speculative to determine how structural changes translate into actual functional recovery. Integrating quantitative strength testing and clinical scoring systems in future research will be essential to validate the prognostic value of US parameters and to fully elucidate the functional significance of tendon regeneration patterns.

Another relevant limitation concerns the variability in surgical techniques. The procedures were performed by multiple surgeons, which may have introduced heterogeneity in harvesting methods—such as differences in tendon stripping length, preservation of distal insertions, or handling of fascial tissue. These technical nuances could directly influence the extent and pattern of tendon regeneration, as well as the degree of MTJ retraction and muscle hypotrophy observed at follow-up. Standardizing the surgical approach or documenting intraoperative details more precisely could help clarify how procedural variability affects regenerative outcomes in future studies.

All ultrasound assessments were performed by a single experienced operator, which ensured internal consistency and minimized measurement variability. However, this study design precluded the calculation of intra- and inter-observer reliability indices (ICC), representing a methodological limitation.

## 6. Conclusions

High-resolution US is a valuable and non-invasive tool for assessing ST tendon regeneration following ACL reconstruction. Due to its real-time imaging capabilities, it allows for a detailed evaluation of the NT morphology, structure, and functional characteristics. It also enables distinguishing between fibrillar and non-fibrillar patterns, which can provide important clues about tissue quality. However, despite its strong diagnostic potential, US has certain limitations. Its interpretation can be operator-dependent, and US cannot be a substitute for histology or give precise functional information of the muscular–tendon unit. US provides only indirect information about the biomechanical properties of the tendon, which should be supplemented with clinical and functional assessments such as strength testing, pain evaluation, and performance metrics.

## Figures and Tables

**Table 1:** Clinical and sonographic data for patients. *YAS* indicates years after surgery. *NT echo*, neotendon echostructure: f is fibrillar-like, whereas nf is non-fibrillar. Hypo, hypoechoic; iso/hyper, isoechoic/hyperechoic (see text). *NT attach*: attachment of the NT. *NT gliding*, NT gliding present (yes) or absent (no). *MTJ shift*, myotendinous junction proximal shift (with respect to the healthy side). *MTJ scarring*, echostructural alteration of the myotendinous junction (see text). *M CSA* %, ratio of the CSA of the semitendinosus muscle between the operated and the healthy side (see text).

Pt	Sex	Age	YAS	NT Echo	NT Thickness (mm)	T Thickness (mm)	NT/T Ratio	NT Attach	NT Gliding	MTJ Shift (cm)	MTJ Scarring	M CSA %
1	M	34	6	nf hypo	2.5	2.2	114%	MHG	yes	6	2	73%
2	M	21	3	f hypo	2.8	2.2	127%	MHG	yes	5	2	70%
3	M	30	3	nf hypo	2.3	2.3	100%	MHG	yes	6	2	61%
4	M	45	4	nf iso/hyper	1.8	2.6	69%	MHG	yes	11	3	45%
5	M	31	5	nf hypo	3	2.2	136%	MHG	yes	6	3	50%
6	M	20	2	f hypo	3.4	2.4	138%	MHG	yes	4	1	87%
7	M	21	4	f hypo	6.4	2.2	291%	Tibia	yes	2	1	100%
8	M	34	6	nf iso/hyper	2.3	2.3	100%	MHG	yes	7	3	51%
9	M	21	4	/	no	2.5	/	/	/	10	3	45%
10	F	48	5	/	no	2.2	/	/	/	12	3	35%
11	M	30	4	f hypo	4.3	2.1	204%	MHG	yes	4	1	81%
12	M	70	6	nf iso/hyper	2.9	2.7	107%	MHG	yes	8	3	57%
13	M	34	6	f hypo	5.7	3.1	184%	Tibia	yes	3	1	96%
14	M	48	2	f hypo	5.4	2.4	225%	MHG	yes	6	3	73%
15	M	50	4	/	no	2.2	/	/	/	10	3	45%
16	M	48	7	nf iso/hyper	4.5	2.6	173%	SM	no	12	3	50%
17	M	26	4	f hypo	3.9	3.1	126%	MHG	yes	5	2	77%
18	F	26	2	f hypo	2.8	2.5	112%	MHG	yes	4	2	81%
19	M	20	6	f hypo	1.6	2.2	72%	MHG	yes	4	2	80%
20	M	29	4	f iso/hyper	3.3	2.4	138%	Tibia	yes	3	2	89%
21	F	36	8	/	no	2.3	/	/	/	12	3	41%
22	M	26	6	f hypo	4.1	2.3	178%	MHG	yes	3	1	98%
23	M	29	5	nf iso/hyper	1.6	2.2	72%	MHG	yes	6	3	57%
24	M	24	3	/	no	2.3	/	/	/	11	3	38%

**Table 2:** Spearman’s correlations exploring morphometrical and clinical parameters.

Variable Pair	Ρ (Spearman)	*p*	*p* (FDR)	Interpretation
MTJ shift ↔ M CSA %	−0.96	<0.001	<0.001	Strong inverse correlation: greater MTJ displacement associated with lower muscle CSA
Age ↔ MTJ shift	+0.57	0.004	0.013	Older age associated with greater MTJ displacement
Age ↔ M CSA %	−0.45	0.029	0.057	Trend toward lower muscle CSA with increasing age
YAS ↔ MTJ shift	+0.22	0.294	0.471	Not significant
YAS ↔ M CSA %	−0.14	0.500	0.666	Not significant

**Table 3:** Difference in the CSA ratio in the two subgroups. The first group includes patients with fibrillar-like NT, whereas the second patients with non-fibrillar NT.

Group	Patients (No.)	CSA Ratio Range (%)	Average CSA Ratio (%)
Fibrillar-like NT	11 (61%)	70–100	77
Non-fibrillar NT	7 (39%)	45–73	57

## Data Availability

The original contributions presented in this study are included in the article/Supplementary Material. Further inquiries can be directed to the corresponding author.

## Supplementary Materials

The following supporting information can be downloaded at https://www.mdpi.com/article/10.3390/jcm14217862/s1, Video S1: Movie S1.
